# Modern Sociology

**Published:** 1902-11-08

**Authors:** 


### Modern Sociology.

### BLACK SMOKE.

Sheffield enjoys an unenviable notoriety as a smoky town, and it is therefore natural that its Medical Officer of Health should protest against the too general opinion that those who live there are indifferent to the factory smoke which is so intimately connected with the wealth—though it may militate against the health—of the town. This Dr. J. Robertson, Medical Officer of Health for Sheffield, does in the September number of "Public Health." The powers for dealing with the smoke created in the process of any manufacture are obtained from the Public Health Act of 1875, and give local authorities power to proceed against "any fireplace or chimney which does not as far as practicable consume the smoke arising from the combustible used therein," and again against "any chimney (not being the chimney of a private dwelling-house) sending forth black smoke in such quantity as to be a nuisance."

It is not usual to prosecute under the first of these clauses, for, as Dr. Robertson says, "Our experience goes to show that with careful firing nearly all boiler furnaces can be fired so as to give off so little black smoke that no nuisance arises." On this assumption—namely, that all the boiler furnaces are so constructed that no nuisance need arise from them—all prosecutions are taken under the second clause which we have quoted. It is taken for granted that when a smoke nuisance arises it is due rather to carelessness in firing than to defects in construction of the furnaces. "Nuisance" is one of the useful words which cannot be accurately measured. What is a nuisance in one case is not a nuisance in another. The same volume of smoke issuing from a low chimney may be a great nuisance to dwellers in the neighbourhood which would occasion little inconvenience if it came from a high one. What is injurious to health in a crowded neighbourhood or in a sheltered valley would be but a trifling disagreeable in a sparsely populated district or on a hill-top. What constitutes a nuisance must be judged by all the circumstances surrounding it.

With regard even to the phrase "black smoke" trouble has arisen. When prosecutions have been entered into, counsel for the defence has often pleaded that the smoke complained of was not "black" in the strict acceptance of the word, but brown or grey. But these subtleties are not allowed to pass. Black smoke is what in common parlance is called black smoke, even as a black gown remains a black gown although its owner may declare that sun and air have turned it to a rusty brown or even a greenish tint. The question is how much of this smoke must be permitted if the trade of Sheffield is to go on.

It has been decided to fix a time-limit for each chimney. The greater the number of boilers, the greater the amount of black smoke allowed per hour. In many towns a time-limit of five or six minutes per hour is fixed, regardless of the number of boilers dealt with. In Sheffield things are analysed more closely. Thus, one boiler is allowed the emission of two minutes' black smoke hourly, two boilers may have three minutes, three boilers four, and four or more boilers six minutes in the hour. Dr. Robertson thinks, however, that without inconveniencing any manufacturers it would be possible to take a minute per hour off each of these allowances. At lighting-up time some allowance must be made. It is impossible for even the most careful fireman entirely to avoid making smoke when lighting his fires, but after that a good deal depends on the fuel employed and the skill in firing. Boilers which burn coke, or coke mixed with only a little coal, should give off no black smoke, or almost none, especially if the firing is done regularly and continuously. Dr. Robertson says "so far as steam-boiler furnaces are concerned, I am sure that it could be done away with, but our trade would soon disappear, as our local coal could not be used, so far as we know at present." With foreign competition to face, it is not likely that Sheffield manufacturers, or any others, will go to the expense of importing anthracite coal for their furnaces. Better methods of stoking, automatic or otherwise, and the judicious use of the fuel of every kind at our command must be relied on. And the sanitary authorities have to take many things into consideration before they enter into a prosecution because of the emission of black smoke from a chimney. When a case of nuisance arising from black smoke is reported, the premises in question are carefully watched by three inspectors, and on their combined reports action is taken. Sometimes a formal notice is sent merely requesting that more care should be taken in firing, and occasionally a demand is added that the chimney should be raised or that coke should be used as fuel. If these requirements are complied with for six months, the notice lapses, but if they are ignored a summons may be taken out, and a fine imposed. "In the matter of fines for smoke nuisances," says Dr. Robertson, "I think we are far too lenient. Frequently a nominal penalty only is imposed, and an order made for the abatement of the nuisance." The maximum penalty for disobedience of a magistrate's order is only £1 a day, which in a big factory is no great thing.

Unfortunately there are some factories to the success of which a cloud of smoke is essential. Metallurgical furnaces are specially exempted from the action of that clause of the Public Health Act which deals with smoke. "Nothing in this Act shall be construed to extend to mines of different descriptions so as to interfere with or obstruct the efficient working of the same, nor to the smelting of ores or minerals, nor to the calcinary puddling and rolling of iron and other metals." The reason for this exemption is that in the manufacture and after-manipulation of steel great care has to be taken to prevent the steel losing its carbon. The heating of the ingots of metal preparatory to rolling or forging requires a slow even heat so that there may be no uneven expansion. This heating is done in a slow, smoky furnace, with what is called an "envelope" of smoke round the ingots, and, moreover, to keep the fire slow it is found necessary to have only a low chimney, as a high one causes too great a draught and too strong a fire. Thus, the smoke which might be done away with in other manufacturing processes seems to be still essential to the working of metals. Under these circumstances it is to be feared that while Sheffield flourishes it will still have to wear its cloudy mantle.

